# Mathematical Modeling of SARS-CoV-2 Transmission between Minks and Humans Considering New Variants and Mink Culling

**DOI:** 10.3390/tropicalmed8080398

**Published:** 2023-08-03

**Authors:** Mahmoud A. Ibrahim, Attila Dénes

**Affiliations:** 1 Bolyai Institute, University of Szeged, Aradi Vértanúk Tere 1., 6720 Szeged, Hungary; 2 Department of Mathematics, Faculty of Science, Mansoura University, Mansoura 35516, Egypt; 3 National Laboratory for Health Security, Bolyai Institute, University of Szeged, Aradi Vértanúk Tere 1., 6720 Szeged, Hungary

**Keywords:** COVID-19, SARS-CoV-2, human-to-mink transmission, mink-to-human transmission, SEIR and SVEIR compartmental models, reproduction number, virus mutation, culling and vaccination strategies

## Abstract

We formulated and studied mathematical models to investigate control strategies for the outbreak of the disease caused by SARS-CoV-2, considering the transmission between humans and minks. Two novel models, namely SEIR and SVEIR, are proposed to incorporate human-to-human, human-to-mink, and mink-to-human transmission. We derive formulas for the reproduction number $R_{0}$ for both models using the next-generation matrix technique. We fitted our model to the daily number of COVID-19-infected cases among humans in Denmark as an example, and using the best-fit parameters, we calculated the values of $R_{0}$ to be 1.58432 and 1.71852 for the two-strain and single-strain models, respectively. Numerical simulations are conducted to investigate the impact of control measures, such as mink culling or vaccination strategies, on the number of infected cases in both humans and minks. Additionally, we investigated the possibility of the mutated virus in minks being transmitted to humans. Our results indicate that to control the disease and spread of SARS-CoV-2 mutant strains among humans and minks, we must minimize the transmission and contact rates between mink farmers and other humans by quarantining such individuals. In order to reduce the virus mutation rate in minks, culling or vaccination strategies for infected mink farms must also be implemented. These measures are essential in managing the spread of SARS-CoV-2 and its variants, protecting public health, and mitigating the potential risks associated with human-to-mink transmission.

## 1. Introduction

Coronaviruses (CoV) are a broad family of viruses, with symptoms that vary from those of the common cold to those of more serious diseases, e.g., Middle East Respiratory Syndrome (MERS-CoV) and Severe Acute Respiratory Syndrome (SARS-CoV). Several coronaviruses can spread between animals and humans, which means they are zoonotic viruses. According to Haider et al. [1], COVID-19 should be classified as an “emerging infectious disease (EID) of probable animal origin”. Based on detailed investigations, SARS-CoV was transmitted from civet cats to humans, whereas MERS-CoV was spread from camels to humans [2,3,4]. Furthermore, several coronaviruses that have not yet infected humans have been identified in animals.

**Figure 1:**
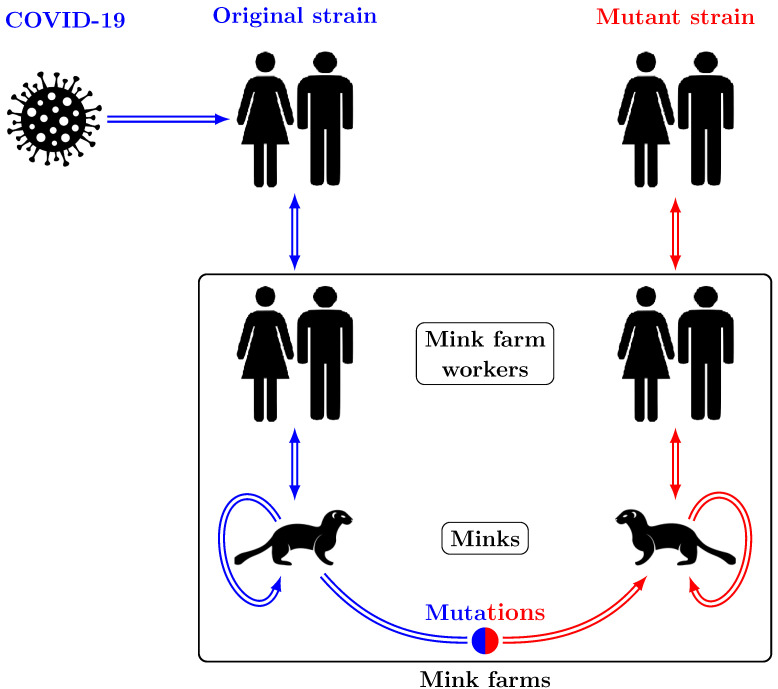
SARS-CoV-2 virus transmission between minks and humans. Blue arrows show the directions of human-to-human, human-to-mink, and mink-to-mink transmission due to the original strain of the virus. Red arrows show mink-to-mink, mink-to-human, and human-to-human transmission due to the mutant strain.

Coronavirus disease 2019 (COVID-19) is a contagious disease caused by the SARS-CoV-2 virus [5]. The disease spreads mainly through human-to-human transmission; however, there have been several reports of disease spread between humans and some animals as well. SARS-CoV-2 ribonucleic acid (RNA) has been identified in animals that have had contact with infected humans, such as owners, caregivers, or anyone who came into close contact with the animals. Animals infected with the virus have been documented all over the world, including minks on mink farms such as American mink (*Neogale vison*), dogs, domestic cats, hyenas, snow leopards, lions, tigers, a binturong, raccoon dogs, non-human primates, otters, a fishing cat, hippopotamuses, a coatimundi, manatees, a giant anteater, white-tailed and mule deer, a black-tailed marmoset, and wild mink near mink farms [6,7,8,9,10,11,12,13,14]. However, thus far, animal-to-human transmission has been observed in the cases of farmed mink in Europe and the US, pet hamsters in Hong Kong, white-tailed deer in Canada, and a cat in Thailand [6,12,15,16,17]. To the best of our knowledge, the World Organisation for Animal Health (WOAH) has received reports of SARS-CoV-2 in farmed mink from the following countries: Denmark, the Netherlands, France, Latvia, Lithuania, Poland, Greece, Italy, Spain, Sweden, Canada, and the USA [12,18,19,20,21]. The possibility of human-to-mink and mink-to-human transmission has also been established [12,15,16,17]. The modes of SARS-CoV-2 virus transmission between minks and humans are presented in Figure 1.

Denmark is the world’s largest mink producer, and the country has 1500 farms that produce mink skins valued at EUR 1.1 billion [22]. In the summer of 2020, a farm in the Danish region of North Jutland announced the first case of COVID-19 infection in farmed mink in Denmark [23]. Despite the fact that the animals were symptom-free, three of the first farms to be identified were culled [22,24]. Since the decision to cull all minks in Denmark to prevent infection to humans was announced on 4 November 2020, over 17 million minks have been culled [25]. About 25% of farms were infected with COVID-19 during the first cull period [15]. Denmark’s authorities commanded a provisional ban on mink farming in December 2020 (initially, until the end of 2021, and then later extended until the end of 2022) [13]. Given the way mink farming was discontinued, the unclear situation surrounding the pandemic’s course, and the efforts of animal rights activists, it seems doubtful that mink farming in Denmark will be able to return to its full potential once the COVID-19 epidemic is under control [22].

A considerable number of scientific works have appeared that assess the epidemiological characteristics of COVID-19 in order to reduce its burden on public health (e.g., [26,27,28,29,30]). Rasmussen et al. [31] developed an SEIRS model that included deaths outside of hospitals, as well as independent assessments of cases with and without symptoms, with varied immune memories. The model was adjusted to account for the progression of the epidemic observed in Denmark. According to the findings, COVID-19 has a low mortality rate since most of the infected individuals are either symptom-free or have mild symptoms. As a result, only a small number of affected people require hospitalization. Valentin et al. [32] used an SEIR-type model to identify the basic reproduction number of the epidemic in Denmark prior to and following the implementation of lockdowns, revealing a considerable drop from 3.32 to 0.92. The lockdown, which began on 18 March 2020, had an effect after a few days. Gumel [33] established a deterministic two-strain model for the dynamics of the transmission of bird flu between birds and humans. The model included the spread of an avian strain and its mutant (which can be transmitted among humans), as well as the isolation of those with symptoms from either strain. The reproduction number determines the system’s global dynamics. Numerical simulations suggested that the disease burden increases as the avian strain’s mutation rate increases. Agusto [34] improved the model of Gumel [33] by adding control over the isolation rate of humans infected with avian and mutant strains. Rashkov and Kooi [35] developed a host-vector model for dengue fever, considering two strains of the virus, allowing temporary cross-immunity for the hosts, and the possibility of secondary infections. Royce and Fu [36] presented a model for transmission among three species that accounts for a zoonotic disease, which mutates in an intermediate host. They found that with realistic parameters of interspecies transmission, a zoonosis with the ability to mutate in an intermediate host species can establish itself in humans, even if the basic reproduction number in humans is lower than 1. Sardar et al. [37] developed three different two-strain MERS-CoV models that take into account human-to-human transmission in the community and hospitals, as well as passive zoonotic transmission, to predict past outbreaks from 2012 to 2016 and obtain key epidemiological information for the following Saudi cities: Mecca, Medina, and Riyadh. They examined infection variability using disease incidence functions of three different forms, capturing social behavior triggered by an epidemic. In their recent research de León et al. [38], a new mathematical model was proposed to account for two virus strains, along with a vaccination program. By applying this model to the pandemic in the United States, the authors accurately forecasted the rise of the alpha variant and highlighted the possible impact of the delta variant in the year 2021. Additionally, they determined the lowest percentage of the fully vaccinated population required, along with other intervention strategies, to effectively reduce the spread of the variants and mitigate the multi-strain pandemic. Tchoumi et al. [39] developed a mathematical model to analyze the transmission dynamics of COVID-19, considering different strains and vaccination effects. The model demonstrated stability and identified the conditions for strain persistence and dominance. Strains would persist if their reproductive numbers exceeded 1. Strain 2 could become dominant if its reproductive number surpassed strain 1’s or if strain 1’s reproductive number was below 1. However, strain 2 would not establish itself if strain 1’s vaccination generated herd immunity and the transmission threshold for strain 2 remained low. Several studies have investigated the COVID-19 pandemic using two-strain models [40,41,42,43].

In this work, we establish two mathematical models to study the COVID-19 outbreak in Denmark, taking into consideration human-to-human, human-to-mink, and mink-to-human transmission. The human population is partitioned into two groups based on the individuals’ contact with minks: humans in direct contact with minks and humans in indirect contact with minks. We construct a two-strain compartmental model by considering the virus mutation in the mink population, as well as the spread of the new SARS-CoV-2 mink variant to humans. Also, we consider a single-strain model by neglecting the virus mutation in minks and, in view of the ongoing development of vaccines for animals, we include a mink vaccination compartment. The purpose of this research is to assess the possibility of the human population being infected by a new mutant virus originating from minks and study the effect of different control measures, such as mink culling or vaccination, on disease transmission between humans and minks. Using numerical simulations, we estimate the parameters of both models using data on the daily number of COVID-19-infected cases among humans in Denmark in order to obtain the best investigation strategies and sensitivity analysis.

## 2. Methods

### 2.1. A Two-Strain Compartmental Model

We developed a dynamic two-strain compartmental model to study the dynamics of the transmission of COVID-19 between human and mink populations. We take into account the SARS-CoV-2 outbreak in Denmark, taking into account human-to-human, human-to-mink, and mink-to-human transmission [12,23]. The total human population is divided into two main sub-populations in our model, depending on their level of contact with minks: those with indirect contact, i.e., those who do not normally come into contact with minks, and those with direct contact, i.e., those who have contact with minks, such as people who work in mink farming. However, we are seeking to account for the possibility of disease mutation in mink populations, as well as the spread of the newly mutated virus among humans [12,14,19,23]. We start by simply extending an SEIR-based model to include two disease strains for each group of human and mink populations.

Compartments for humans with indirect contact, with direct contact, and minks, are denoted by the lower indices u,d, and *m*, respectively. We consider three populations (i=u,d, or *m*) with two strains of the SARS-CoV-2 virus (denoted by the index j=1 or j=2). In this compartmental model, for each population, susceptible ($S_{i}$) classes are those who can be infected by the SARS-CoV-2 virus. After contracting the disease, one enters the exposed class ($E_{i_{j}}$), which takes into account those who do not yet show any symptoms. Exposed individuals enter the corresponding infectious class ($I_{i_{j}}$) once they become infected by either strain of the disease, and then they transition to the corresponding recovered class ($R_{i}$) after the infection period. $D_{i}$ represents the death compartment for each sub-population. We assume that the virus mutates in minks at rate γ, and hence the newly mutated virus is transmitted to the human populations. Individuals may also leave any of the infected compartments ($I_{i_{j}}$) through disease-induced death through rates ($\delta_{1},\delta_{2},\delta_{3}$, and $\delta_{4}$).

We use the notation $N_{u}\left(t\right)$ for the total human population that has indirect contact with minks, $N_{d}\left(t\right)$ for the total human population that has direct contact with minks, and $N_{m}\left(t\right)$ for the total mink population, which is given by:

$$\begin{array}{cc} N_{u}\left(t\right)= & S_{u}\left(t\right)+E_{u_{1}}\left(t\right)+E_{u_{2}}\left(t\right)+I_{u_{1}}\left(t\right)+I_{u_{2}}\left(t\right)+R_{u}\left(t\right)+D_{u}\left(t\right),\\ N_{d}\left(t\right)= & S_{d}\left(t\right)+E_{d_{1}}\left(t\right)+E_{d_{2}}\left(t\right)+I_{d_{1}}\left(t\right)+I_{d_{2}}\left(t\right)+R_{d}\left(t\right)+D_{d}\left(t\right),\\ N_{m}\left(t\right)= & S_{m}\left(t\right)+E_{m_{1}}\left(t\right)+E_{m_{2}}\left(t\right)+I_{m_{1}}\left(t\right)+I_{m_{2}}\left(t\right)+R_{m}\left(t\right)+D_{m}\left(t\right). \end{array}$$

**Figure 2:**
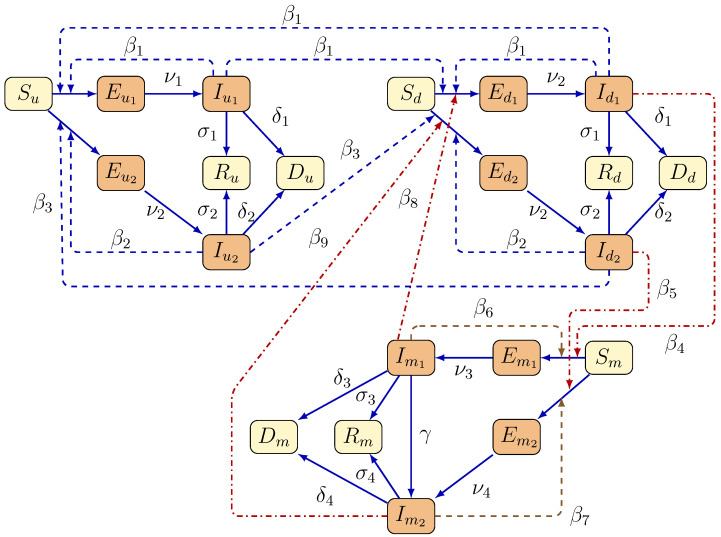
Transmission dynamics of a two-strain mathematical model for the spread of the SARS-CoV-2 between minks and humans. The three sub-populations are indicated by lower indices: Humans with indirect contact (*u*), humans with direct contact (*d*), and mink compartments (*m*). The compartments S, E, I, R, and *D* represent susceptible, exposed, infected, recovered, and death, respectively. Brown nodes indicate infectious compartments, whereas yellow nodes indicate non-infectious compartments. Blue solid arrows represent disease progression. Dashed blue arrows represent transmission among humans. Red dashed lines represent human-to-mink and mink-to-human transmission, and brown dashed lines show mink-to-mink transmission.

The transmission dynamics of the model are illustrated in Figure 2.

Based on the transmission chart shown in Figure 2 and the summary of parameters listed in Table 1, the corresponding system of differential equations takes the form

(1) $$\begin{array}{cc} S_{u}^{\prime }= & -\frac{\beta_{1}I_{u_{1}}+\beta_{2}I_{u_{2}}}{N_{u}}S_{u}-\frac{\beta_{1}I_{d_{1}}+\beta_{3}I_{d_{2}}}{N_{d}}S_{u},\\ E_{u_{1}}^{\prime }= & \beta_{1}\frac{I_{u_{1}}}{N_{u}}S_{u}+\beta_{1}\frac{I_{d_{1}}}{N_{d}}S_{u}-\nu_{1}E_{u_{1}},\\ I_{u_{1}}^{\prime }= & \nu_{1}E_{u_{1}}-\sigma_{1}I_{u_{1}}-\delta_{1}I_{u_{1}},\\ E_{u_{2}}^{\prime }= & \beta_{2}\frac{I_{u_{2}}}{N_{u}}S_{u}+\beta_{3}\frac{I_{d_{2}}}{N_{d}}S_{u}-\nu_{2}E_{u_{2}},\\ I_{u_{2}}^{\prime }= & \nu_{2}E_{u_{2}}-\sigma_{2}I_{u_{2}}-\delta_{2}I_{u_{2}},\\ R_{u}^{\prime }= & \sigma_{1}I_{u_{1}}+\sigma_{2}I_{u_{2}},\\ D_{u}^{\prime }= & \delta_{1}I_{u_{1}}+\delta_{2}I_{u_{2}},\\ S_{d}^{\prime }= & -\frac{\beta_{1}I_{d_{1}}+\beta_{2}I_{d_{2}}}{N_{d}}S_{d}-\frac{\beta_{1}I_{u_{1}}+\beta_{3}I_{u_{2}}}{N_{u}}S_{d}-\frac{\beta_{8}I_{m_{1}}+\beta_{9}I_{m_{2}}}{N_{m}}S_{d},\\ E_{d_{1}}^{\prime }= & \beta_{1}\frac{I_{d_{1}}}{N_{d}}S_{d}+\beta_{1}\frac{I_{u_{1}}}{N_{u}}S_{d}+\beta_{8}\frac{I_{m_{1}}}{N_{m}}S_{d}-\nu_{1}E_{d_{1}},\\ I_{d_{1}}^{\prime }= & \nu_{1}E_{d_{1}}-\sigma_{1}I_{d_{1}}-\delta_{1}I_{d_{1}},\\ E_{d_{2}}^{\prime }= & \beta_{2}\frac{I_{d_{2}}}{N_{d}}S_{d}+\beta_{3}\frac{I_{u_{2}}}{N_{u}}S_{d}+\beta_{9}\frac{I_{m_{2}}}{N_{m}}S_{d}-\nu_{2}E_{d_{2}},\\ I_{d_{2}}^{\prime }= & \nu_{2}E_{d_{2}}-\sigma_{2}I_{d_{2}}-\delta_{2}I_{d_{2}},\\ R_{d}^{\prime }= & \sigma_{1}I_{d_{1}}+\sigma_{2}I_{d_{2}},\\ D_{d}^{\prime }= & \delta_{1}I_{d_{1}}+\delta_{2}I_{d_{2}},\\ S_{m}^{\prime }= & \Lambda -\frac{\beta_{4}I_{d_{1}}+\beta_{5}I_{d_{2}}}{N_{d}}S_{m}-\frac{\beta_{6}I_{m_{1}}+\beta_{7}I_{m_{2}}}{N_{m}}S_{m}-\mu S_{m},\\ E_{m_{1}}^{\prime }= & \beta_{4}\frac{I_{d_{1}}}{N_{d}}S_{m}+\beta_{6}\frac{I_{m_{1}}}{N_{m}}S_{m}-\nu_{3}E_{m_{1}}-\mu E_{m_{1}},\\ I_{m_{1}}^{\prime }= & \nu_{3}E_{m_{1}}-\gamma I_{m_{1}}-\sigma_{3}I_{m_{1}}-\left(\delta_{3}+\mu \right)I_{m_{1}},\\ E_{m_{2}}^{\prime }= & \beta_{5}\frac{I_{d_{2}}}{N_{d}}S_{m}+\beta_{7}\frac{I_{m_{2}}}{N_{m}}S_{m}-\nu_{4}E_{m_{2}}-\mu E_{m_{2}},\\ I_{m_{2}}^{\prime }= & \nu_{4}E_{m_{2}}+\gamma I_{m_{1}}-\sigma_{4}I_{m_{2}}-\left(\delta_{4}+\mu \right)I_{m_{2}},\\ R_{m}^{\prime }= & \sigma_{3}I_{m_{1}}+\sigma_{4}I_{m_{2}}-\mu R_{m},\\ D_{m}^{\prime }= & \delta_{3}I_{m_{1}}+\delta_{4}I_{m_{2}}. \end{array}$$

We denote by Λ the mink birth rate and by μ the mink death rate. It is worth noting that minks are only born in April and May each year. Since we studied the COVID-19 pandemic in Denmark during the period from 1 September 2020 to 1 March 2021, we set the mink birth rate (Λ) to zero. The terms $\beta_{i},\;i=1,\cdots ,9,$ represent the transmission rates. Specifically, $\beta_{i},\;i=1,\cdots ,3,$ are the human-to-human transmission rates, whereas $\beta_{4}$ and $\beta_{5}$ are the human-to-mink transmission rates. Minks transmit the disease to humans at rates $\beta_{6}$ and $\beta_{7}$, whereas the mink-to-mink transmission rates are $\beta_{8}$ and $\beta_{9}$. The parameter γ is the minks’ mutation rate between the infected classes $I_{m_{1}}$ and $I_{m_{2}}$. The duration of the latent period for humans is $1/\nu_{1},\;1/\nu_{2}$, whereas $1/\nu_{3},\;1/\nu_{4}$, is the duration of the latent period for minks. We denote the duration of the infected period for infected humans by $1/\sigma_{1},\;1/\sigma_{2}$; the length of the infected period for infected minks by $1/\sigma_{3},\;1/\sigma_{4}$; infected humans’ disease-induced death rates by $1/\delta_{1},\;1/\delta_{2}$; and infected minks’ disease-induced death rates by $1/\delta_{3},\;1/\delta_{4}$. Table 1 describes the variables and parameters used in our work.

### 2.2. A Single-Strain Compartmental Model with Vaccinated Minks

**Figure 3:**
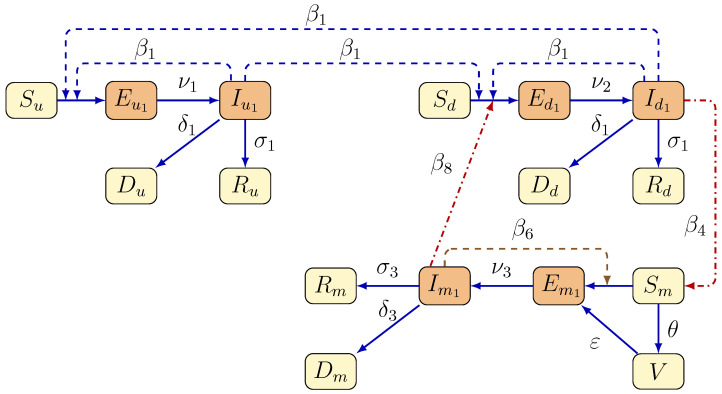
Transmission diagram of the mink vaccination mathematical model for the spread of COVID-19 between humans and minks.

To study the next main question of our work, we also consider a single-strain mathematical model in which the virus mutation rate (γ) in minks is zero. To assess the effect of vaccinations on the number of COVID-19-infected cases in minks and humans, we introduced a new class (*V*) that includes vaccinated minks, where vaccination happens at a rate of θ. Susceptible humans ($S_{u},S_{d}$), after contracting the disease, move to the exposed classes ($E_{u_{1}},E_{d_{1}}$) before becoming infected and entering the infected classes ($I_{u_{1}},I_{d_{1}}$) once they become infectious. Following the infectious period, infected humans proceed to the recovered compartments ($R_{u},R_{d}$) after recovery. The total population of minks is divided into six compartments: susceptible ($S_{m}$), vaccinated (*V*), exposed ($E_{m_{1}}$), infected ($I_{m_{1}}$), and recovered ($R_{m}$). $D_{u}$, $D_{d}$, and $D_{m}$ represent the death compartment for each sub-population. The transmission dynamics of the model are shown in Figure 3.

We reduce Model (1) to a single-strain model in this section by assuming that the virus mutation rate in minks is zero and introducing a new compartment (V(t)) for vaccinated minks. As a result, the total human population with indirect contact with minks ($N_{u}\left(t\right)$), total human population with direct contact with minks ($N_{d}\left(t\right)$), and total mink population ($N_{m}\left(t\right)$) are given by:

$$\begin{array}{cc} N_{u}\left(t\right)= & S_{u}\left(t\right)+E_{u_{1}}\left(t\right)+I_{u_{1}}\left(t\right)+R_{u}\left(t\right)+D_{u}\left(t\right),\\ N_{d}\left(t\right)= & S_{d}\left(t\right)+E_{d_{1}}\left(t\right)+I_{d_{1}}\left(t\right)+R_{d}\left(t\right)+D_{d}\left(t\right),\\ N_{m}\left(t\right)= & S_{m}\left(t\right)+V\left(t\right)+E_{m_{1}}\left(t\right)+I_{m_{1}}\left(t\right)+R_{m}\left(t\right)+D_{m}\left(t\right). \end{array}$$

The transmission modes are displayed in the flow diagram in Figure 3, and the parameter descriptions are shown in Table 1. Our model can be written in the form

(2) $$\begin{array}{cc} S_{u}^{\prime }= & -\beta_{1}\frac{I_{u_{1}}}{N_{u}}S_{u}-\beta_{1}\frac{I_{d_{1}}}{N_{d}}S_{u},\\ E_{u_{1}}^{\prime }= & \beta_{1}\frac{I_{u_{1}}}{N_{u}}S_{u}+\beta_{1}\frac{I_{d_{1}}}{N_{d}}S_{u}-\nu_{1}E_{u_{1}},\\ I_{u_{1}}^{\prime }= & \nu_{1}E_{u_{1}}-\sigma_{1}I_{u_{1}}-\delta_{1}I_{u_{1}},\\ R_{u}^{\prime }= & \sigma_{1}I_{u_{1}},\\ D_{u}^{\prime }= & \delta_{1}I_{u_{1}},\\ S_{d}^{\prime }= & -\beta_{1}\frac{I_{d_{1}}}{N_{d}}S_{d}-\beta_{1}\frac{I_{u_{1}}}{N_{u}}S_{d}-\beta_{8}\frac{I_{m_{1}}}{N_{m}}S_{d},\\ E_{d_{1}}^{\prime }= & \beta_{1}\frac{I_{d_{1}}}{N_{d}}S_{d}+\beta_{1}\frac{I_{u_{1}}}{N_{u}}S_{d}+\beta_{8}\frac{I_{m_{1}}}{N_{m}}S_{d}-\nu_{1}E_{d_{1}},\\ I_{d_{1}}^{\prime }= & \nu_{1}E_{d_{1}}-\sigma_{1}I_{d_{1}}-\delta_{1}I_{d_{1}},\\ R_{d}^{\prime }= & \sigma_{1}I_{d_{1}},\\ D_{d}^{\prime }= & \delta_{1}I_{d_{1}},\\ S_{m}^{\prime }= & \Lambda -\beta_{4}\frac{I_{d_{1}}}{N_{d}}S_{m}-\beta_{6}\frac{I_{m_{1}}}{N_{m}}S_{m}-\theta S_{m}-\mu S_{m},\\ V^{\prime }= & \theta S_{m}-\varepsilon \beta_{6}\frac{I_{m_{1}}}{N_{m}}V-\mu V,\\ E_{m_{1}}^{\prime }= & \beta_{4}\frac{I_{d_{1}}}{N_{d}}S_{m}+\beta_{6}\frac{I_{m_{1}}}{N_{m}}\left(S_{m}+\varepsilon V\right)-\nu_{3}E_{m_{1}}-\mu E_{m_{1}},\\ I_{m_{1}}^{\prime }= & \nu_{3}E_{m_{1}}-\sigma_{3}I_{m_{1}}-\left(\mu +\delta_{3}\right)I_{m_{1}},\\ R_{m}^{\prime }= & \sigma_{3}I_{m_{1}}-\mu R_{m},\\ D_{m}^{\prime }= & \delta_{3}I_{m_{1}}. \end{array}$$

The same parameters as in Model (1) are utilized here, with the addition of two new parameters: the mink vaccination rate (θ) and the baseline value of infected vaccinated minks (ε).

### 2.3. Basic Reproduction Number and Sensitivity Analysis

The basic reproduction number $R_{0}$ is an important threshold parameter for assessing the level of intervention measures necessary to eradicate infectious diseases. This quantity is defined as the expected number of secondary infections generated by a single infected individual in its full infectious period in a population where all other individuals are completely susceptible. We follow the general method of Diekmann et al. [44] and Van den Driessche and Watmough [45] to determine the formula for the basic reproduction number. The derivation of the formula for the basic reproduction number of the two-strain model can be found in Appendix A.

To determine the parameters with the highest effects on the number of infected human cases, we use the Latin hypercube sampling (LHS) method and calculate the partial rank correlation coefficients (PRCCs; see, e.g., Blower and Dowlatabadi [46]) for various input parameters to perform sensitivity analysis. The PRCC-based sensitivity analysis measures how each parameter affects the number of infected human cases when the parameters are changed within the given ranges.

### 2.4. COVID-19 Data from Denmark

**Figure 4:**
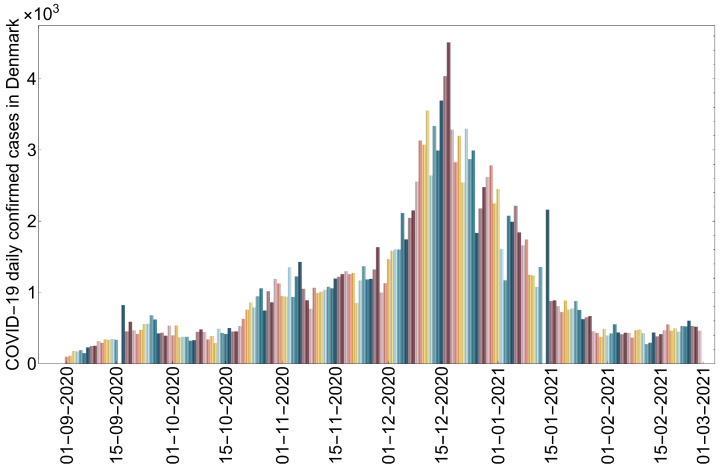
The daily number of infected COVID-19 cases among humans in Denmark from 1 September 2020 to 1 March 2021.

Using data obtained from the Worldometer database [47], we concentrate on the daily number of COVID-19-infected cases among humans from 1 September 2020 to 1 March 2021. Figure 4 shows the daily number of COVID-19-infected cases among humans in Denmark.

## 3. Results

### 3.1. Results Concerning the Two-Strain Model with Mutation

**Figure 5:**
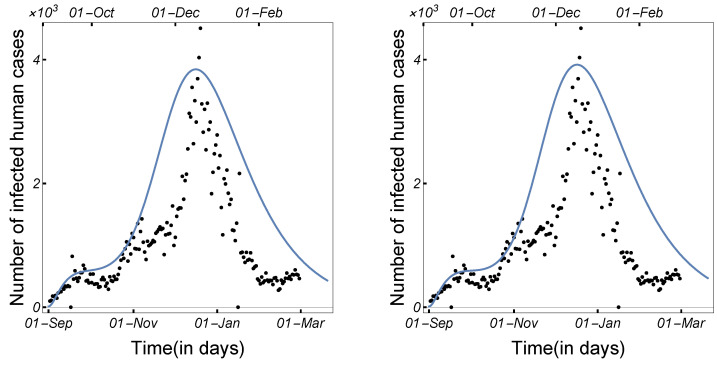
The best-fit solutions of the two-strain model (1) (**left panel**) and single-strain model (2) (**right panel**) to the daily number of COVID-19-infected cases among humans in Denmark, as presented in Figure 4, using the parameters from Table 2.

Our aim in this subsection is to study the possibility that the newly mutated virus invades the human population, as well as to show the impact of culling minks on the spread of the virus in the mink population and hence in the human population. To attain the best results for presenting our idea, we used the system with mutant strains infecting humans and minks and the daily number of COVID-19-infected cases among humans in Denmark (see Figure 4). It is worth noting that minks are only born in April and May each year. Since we studied the COVID-19 pandemic in Denmark during the period from 1 September 2020 to 1 March 2021, we set the mink birth rate (Λ) to zero [48,49,50]. In the figures in this section, the number of daily confirmed COVID-19 infections among humans is represented by a dot. We utilized Latin hypercube sampling, together with the least-squares method, to estimate the parameter values of (1) that yield the best-fitting solution to the daily COVID-19-infected cases among humans presented in Figure 4. This sampling technique was employed to simultaneously evaluate the variability of multiple parameter values (for more information, see [51]). The best-fit parameter values are shown in Table 2, and the best-fit solution was considered as the baseline, as shown in Figure 5. We used the obtained fit to perform numerical simulations and sensitivity analyses to determine how the different parameters affect the number of infected cases, particularly those that may be subject to some control measures. The initial conditions were set as follows: $S_{u}\left(0\right)$ = 5,831,400, $E_{u_{1}}\left(0\right)=100$, $I_{u_{1}}\left(0\right)=10$, $E_{u_{2}}\left(0\right)=0$, $I_{u_{2}}\left(0\right)=0$, $R_{u}\left(0\right)=0$, $S_{d}\left(0\right)=2500,E_{d_{1}}\left(0\right)=10$, $I_{d_{1}}\left(0\right)=1$, $E_{d_{2}}\left(0\right)=0$, $I_{d_{2}}\left(0\right)=0$, $R_{d}\left(0\right)=0$, $S_{m}\left(0\right)$ = 17,000,000, $E_{m_{1}}\left(0\right)=10$, $I_{m_{1}}\left(0\right)=1$, $E_{m_{2}}\left(0\right)=0$, $I_{m_{2}}\left(0\right)=0$, and $R_{m}\left(0\right)=0$.

#### 3.1.1. Impact of Transmission Rates and Incubation Period

**Figure 6:**
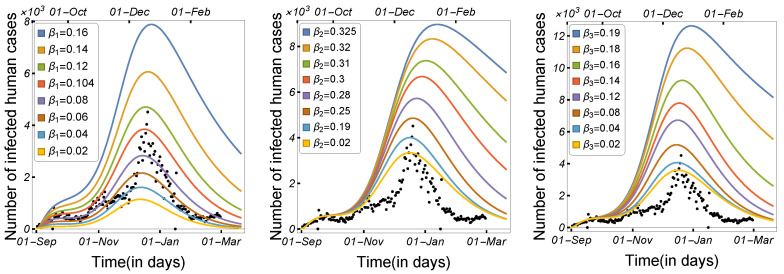
Model (1) solutions (human-infected cases) with respect to human-to-human transmission rates $\beta_{1}$, $\beta_{2}$, and $\beta_{3}$, using the parameters given in Table 2.

**Figure 7:**
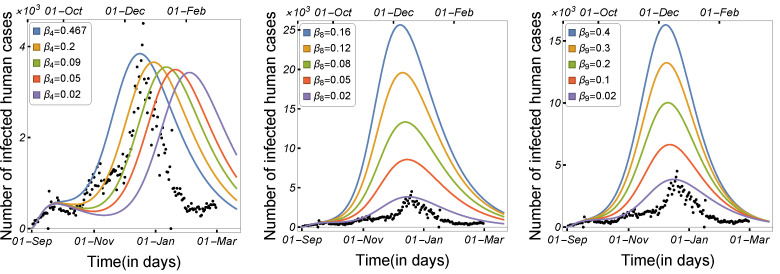
Model (1) solutions (human-infected cases) with respect to the human-to-mink transmission rate ($\beta_{4}$) and mink-to-human transmission rates ($\beta_{8}$ and $\beta_{9}$), using the parameters given in Table 2.

**Figure 8:**
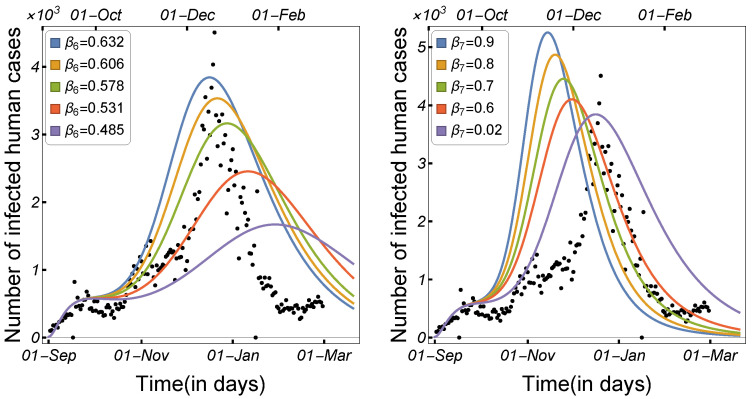
Model (1) solutions (human-infected cases) with respect to mink-to-mink transmission rates $\beta_{6}$, and $\beta_{7}$, using the parameters given in Table 2.

Infectious disease spread is highly affected by the transmission and contact rates, as well as the disease incubation period. As a result, in order to control the disease epidemic, the transmission rates must be reduced to a certain level. We compared and estimated the influence of the transmission modes utilized in our model on the spread of COVID-19 among minks and humans. The rate of human-to-human transmission by either strain, as illustrated in Figure 6, increased the number of infected humans. As presented in Figure 7, decreasing the human-to-mink transmission rate had little effect on decreasing the number of human infections, whereas mink-to-human transmission rates had a large impact on the increase in COVID-19 infections in humans. Mink-to-mink transmission rates by either strain had a significant impact on controlling the outbreak in minks, therefore reducing the number of COVID-19 human infections (see Figure 8).

During an epidemic, knowing the incubation period of an infectious disease can provide important information, such as when infected people will become symptomatic and are most likely to transmit the disease, the severity of the disease, and how long that individual’s illness is likely to last. In the current COVID-19 pandemic, the infectious coronavirus takes between 2 and 14 days to incubate [52,53]. This has significant consequences for disease surveillance and preventive measures like self-quarantine, which should last at least 5 days for everyone who has been exposed to the virus [58].

**Figure 9:**
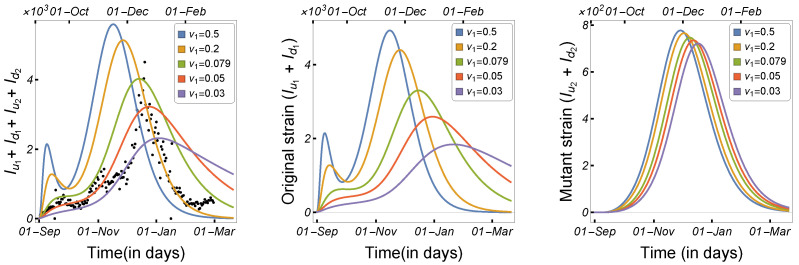
Model (1) solutions (human-infected cases) as a function of the human incubation rate $\nu_{1}$ for (**left panel**) $I_{u_{1}}+I_{d_{1}}+I_{u_{2}}+I_{d_{2}}$, (**middle panel**) $I_{u_{1}}+I_{d_{1}}$, and (**right panel**) $I_{u_{2}}+I_{d_{2}}$, using the parameters given in Table 2.

**Figure 10:**
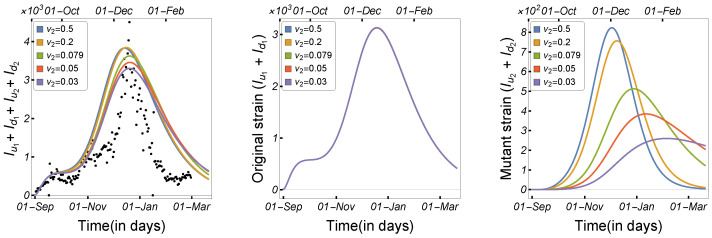
Model (1) solutions (human-infected cases) as a function of the human incubation rate $\nu_{2}$ for (**left panel**) $I_{u_{1}}+I_{d_{1}}+I_{u_{2}}+I_{d_{2}}$, (**middle panel**) $I_{u_{1}}+I_{d_{1}}$, and (**right panel**) $I_{u_{2}}+I_{d_{2}}$, using the parameters described in Table 2.

**Figure 11:**
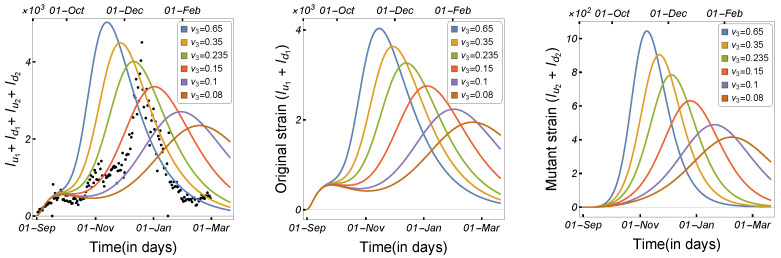
Model (1) solutions (human-infected cases) as a function of the mink incubation rate $\nu_{3}$ for (**left panel**) $I_{u_{1}}+I_{d_{1}}+I_{u_{2}}+I_{d_{2}}$, (**middle panel**) $I_{u_{1}}+I_{d_{1}}$, and (**right panel**) $I_{u_{2}}+I_{d_{2}}$, using the parameters given in Table 2.

In this work, we studied and estimated the impact of the incubation time on the spread of COVID-19 among humans and minks. The total number of human cases and the number of human infections by each strain were plotted against the human incubation rate $\nu_{1}$, as shown in Figure 9. The results indicate that a short incubation period significantly accelerated disease transmission among humans, with $I_{u_{1}}+I_{d_{1}}$ increasing significantly, despite a slight growth in the number of infected people in the mutant strain. Similarly, the number of human cases was plotted against the mutant strain’s incubation rate ($\nu_{2}$), as shown in Figure 10. The results indicate that the incubation period only increased the number of infections in the mutant strain, and thus the total number of infected humans. In Figure 11, the total number of infected individuals is shown against the mink incubation rate $\nu_{3}$. If the disease incubation time in minks was short, the model indicated a highly increased number of infected minks and, therefore, an overall increase in the number of infected humans. The observations indicate that the high mink incubation rate raises $I_{u_{1}}+I_{d_{1}}$ and that, as a result of virus mutation in minks, the number of human infections in the mutant strain increases dramatically.

#### 3.1.2. Is It Possible for the Mutated Virus to Invade the Human Population?

In this subsection, we attempt to answer one of the most important questions in this study: under what conditions and in what situations or scenarios could the new variant virus in minks invade the human population?

**Figure 12:**
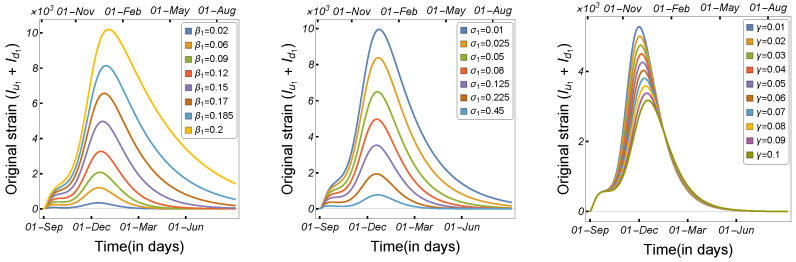
Model (1) solutions (human-infected cases) from the original strain with respect to (**left panel**) human-to-human transmission rate $\beta_{1}$, (**middle panel**) human recovery rate $\sigma_{1}$, and (**right panel**) mutation rate γ, using the parameters described in Table 2.

**Figure 13:**
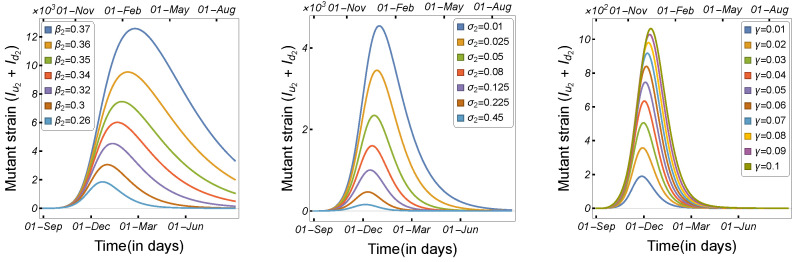
Model (1) solutions (human-infected cases) from the mutant strain with respect to (**left panel**) human-to-human transmission rate $\beta_{2}$, (**middle panel**) human recovery rate $\sigma_{2}$, and (**right panel**) mutation rate γ, using the parameters described in Table 2.

To begin, we illustrate the model solutions’ dependence on the most affected parameters in the transmission of the mutated virus. Figure 12 and Figure 13 show the number of infected humans by each strain with respect to the human-to-human transmission rate, the length of the infection period, and the virus mutation rate in minks. As can be seen, a high transmission rate and a longer infection duration increased the number of human infections in each strain; however, the high mutation of the virus in minks increased the number of infected individuals owing to the mutant strain more than the original strain. Therefore, we prepared simulation scenarios to investigate the types of changes in the parameters that could result in a significant spread of the new variant among human populations.

Scenario 1: Effect of High Transmission Rate with Long Infection or High Mutation Rate

**Figure 14:**
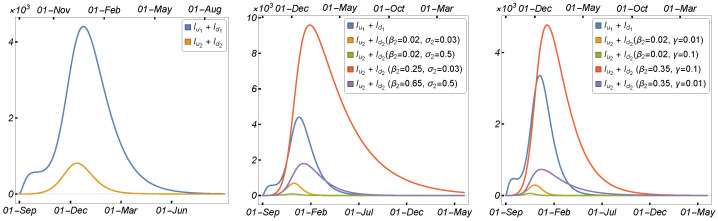
Simulation scenarios using Model (1) to demonstrate the possibility of the mutated virus invading the human population, using the parameters given in Table 2.

We consider the baseline in the left panel of Figure 14, using the parameters given in Table 2. The number of infected humans due to the mutant strain was plotted as a function of the human-to-human transmission rate ($\beta_{2}$) and the recovery rate ($\sigma_{2}$) in the middle panel of Figure 14 and the virus mutation rate (γ) in the right panel of Figure 14. The results indicate that there were only two scenarios in which the mutant strain could invade the original strain (i.e., $I_{u_{2}}+I_{d_{2}}>I_{u_{1}}+I_{d_{1}}$): when the transmission rate was high and the infection period was long, or when the transmission rate and the virus mutation rate were high. This indicates that to control the disease and the spread of the new mutant virus, we must reduce the transmission and contact rates between mink farmers and other humans by quarantining such people in the workplace or, at the very least, in their homes.

Scenario 2: Effect of High Transmission Rates on Human Populations

**Figure 15:**
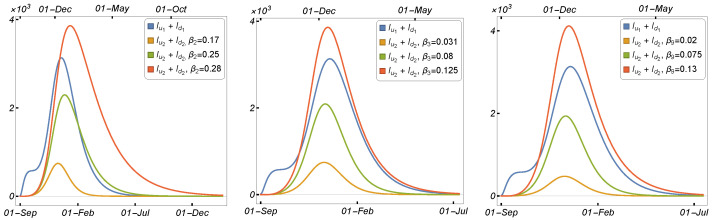
Simulation scenarios using Model (1) to demonstrate the possibility of the mutated virus invading the human population, using the parameters given in Table 2.

In this scenario, the growth in the number of infected humans due to the mutant strain and the mutated virus is primarily attributed to the higher transmission rates. Figure 15 shows the number of infected humans due to the mutant strain plotted as a function of the human-to-human transmission rate ($\beta_{2}$) in the left panel, the human-to-human transmission rate ($\beta_{3}$) in the middle panel, and the mink-to-human transmission rate ($\beta_{9}$) in the right panel. Our findings suggest that a direct increase in the human-to-human transmission rates ($\beta_{2}$ or $\beta_{3}$), as well as the mink-to-human transmission rate ($\beta_{9}$), could result in a significant increase in the number of infected humans affected by the second strain caused by the mutated virus. Hence, the mutant strain has the ability to invade the original strain. This underscores the necessity of controlling the disease and preventing the spread of the new mutant virus by implementing measures to decrease transmission and contact rates between mink and humans, as well as between mink farmers and other individuals. It is crucial to consider quarantining such individuals, either at their workplaces or, at the very least, in their homes.

Scenario 3: Effects of High Transmission and Mutation Rates on Mink Populations

In this scenario, we concentrate on investigating the consequences of specific parameter variations on the spread of the new mink variant within mink populations. We have determined that certain parameters, including the human-to-mink transmission rate ($\beta_{5}$), the mink-to-mink transmission rate ($\beta_{7}$), and the mink mutation rate (γ), had minimal impact on the spread of the new mink variant among human populations. This is due to our assumption that the virus mutation occurred exclusively within mink populations. However, these same parameters exerted a significant influence on the spread of the new mink SARS-CoV-2 variant within mink populations. To illustrate this, we present numerical simulation scenarios that demonstrate the impact of these parameters on the number of infected mink in the second strain.

**Figure 16:**
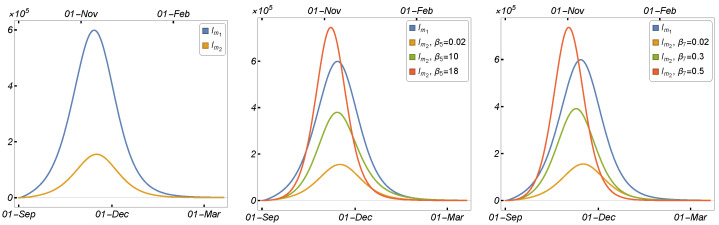
Simulation scenarios using Model (1) to demonstrate the possibility of the mutated virus invading the human population, using the parameters given in Table 2.

**Figure 17:**
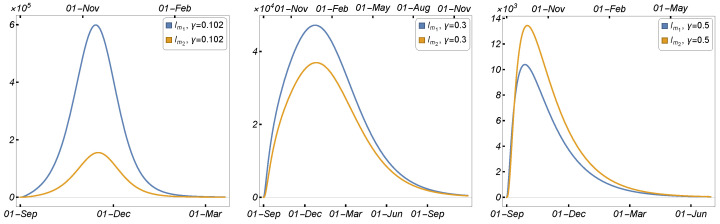
Simulation scenarios using Model (1) to demonstrate the possibility of the mutated virus invading the mink population, using the parameters given in Table 2.

Figure 16 displays the baseline using the parameters specified in Table 2 in the left panel, the number of minks infected due to the mutant strain plotted against the human-to-mink transmission rate ($\beta_{5}$) in the middle panel, and the mink-to-human transmission rate ($\beta_{7}$) in the right panel. Figure 17 illustrates the number of infected mink caused by both the original and mutant strains, plotted as a function of the mink mutation rate (γ). The left panel depicts the baseline using the parameters detailed in Table 2. As we increased the value of γ, we observed the spread of the mutated virus within the mink populations, as demonstrated in the right panel at around γ≈0.5.

This indicates that to control the spread of the new mink variant, it is important to focus on reducing the transmission rates between minks and humans and monitoring and managing the mink mutation rate. Furthermore, implementing culling or vaccination strategies for infected mink farms is necessary to minimize the virus mutation rate in minks. In the rest of this work, we present and discuss the impact of culling minks, as well as the vaccination of minks, on the spread of SARS-CoV-2 among humans and minks.

#### 3.1.3. The Impact of Culling Minks on the Spread of COVID-19

Following the discovery of the COVID-19 virus in minks, several countries have concluded that culling minks is the most effective strategy to control the epidemic and reduce the number of COVID-19 infections in humans, as seen in Denmark. We describe and investigate two simulation scenarios using the two-strain model to show how mink culling affects the spread of COVID-19 among humans and minks.

Scenario 1: Using Three Distinct Culling Ratios on Six Distinct Culling Dates

In this scenario, we evaluate the value of each variable at various time points, considering possible dates for mink culling, using the parameter values listed in Table 2. Following that, we start the new solution by using the set of variables obtained in the first step as the initial conditions and applying three mink culling strategies at six different culling times.

**Figure 18:**
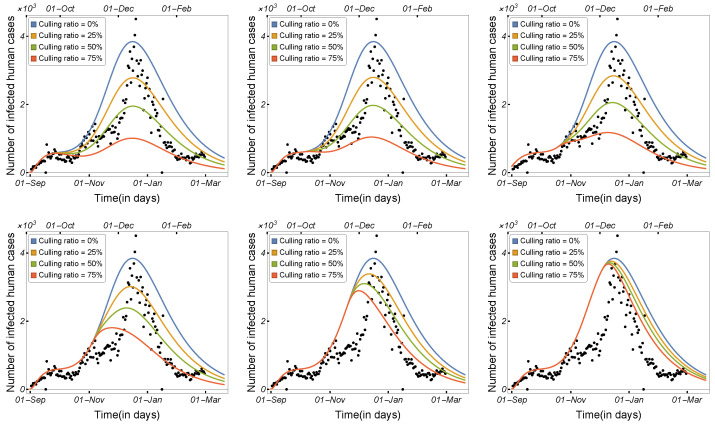
Model (1) solutions (human-infected cases) on the culling dates of 15 September 2020, 1 October 2020, 15 October 2020, 1 November 2020, 15 November 2020, and 1 December 2020, respectively, using the parameters given in Table 2.

Figure 18 depicts the daily number of human-infected cases before and after mink culling, starting with a culling ratio of zero (0%) and continuing until 75% of the total population of minks is culled. The first row in Figure 18 illustrates that the early culling was effective and that the number of infected humans significantly decreased. The overall trend in Figure 18 suggests that the effective culling time was from September 2020 to November 2020. These findings are consistent with Denmark’s decision to cull all minks on 4 November 2020.

Scenario 2: Increasing the Death Rate of Minks

Here, we aim to increase the number of mink deaths and demonstrate how killing minks affects the number of infected humans. Hence, in this scenario, culling appears to add to the minks’ natural death rate. Numerical simulations were prepared in which we plotted the curve of the infected humans using nine different values for the mink death rate.

**Figure 19:**
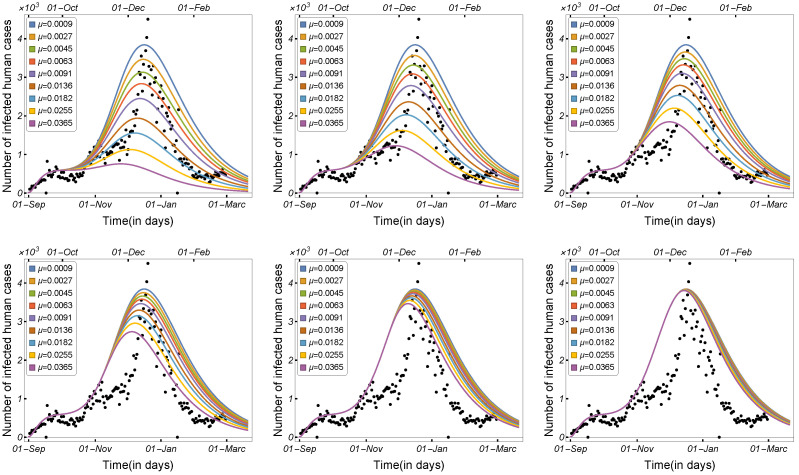
Model (1) solutions (human-infected cases) with respect to the mink death rate on the culling dates of 15 September 2020, 1 October 2020, 15 October 2020, 1 November 2020, 15 November 2020, and 1 December 2020, respectively, using the parameters given in Table 2.

The daily number of infected humans was plotted at six different times, as shown in Figure 19. Increasing the number of mink deaths in September and October resulted in a significant decrease in the number of COVID-19 cases in humans (see the first row of Figure 19), whereas increasing the number of mink deaths in November and December had a smaller impact, as shown in the second row in Figure 19.

### 3.2. Results for the Single-Strain Model with Vaccination in Minks

In this subsection, we aim to study the impact of mink vaccination on disease transmission between humans and minks using the single-strain model, as well as the impact of culling minks in comparison to the results obtained in the previous subsection.

#### 3.2.1. The Impact of Mink Vaccination on COVID-19 Transmission

The single-strain model is used here to illustrate how mink vaccination affects the number of infections in humans and minks. Our strategy was based on the mink vaccination rate, starting with θ=0, which means that no vaccine was introduced, and increasing θ to a certain value to increase the number of vaccinated minks. The initial conditions were as follows: $S_{u}\left(0\right)$ = 5,831,400, $E_{u_{1}}\left(0\right)\left(0\right)=100$, $I_{u_{1}}\left(0\right)=10$, $R_{u}\left(0\right)=0,S_{d}\left(0\right)=2500$, $E_{d_{1}}\left(0\right)=10$, $I_{d_{1}}\left(0\right)=1,R_{d}\left(0\right)=0$, $S_{m}\left(0\right)$ = 17,000,000, V(0)=0, $E_{m_{1}}\left(0\right)=10$, $I_{m_{1}}\left(0\right)=1$, and $R_{m}\left(0\right)=0.$

**Figure 20:**
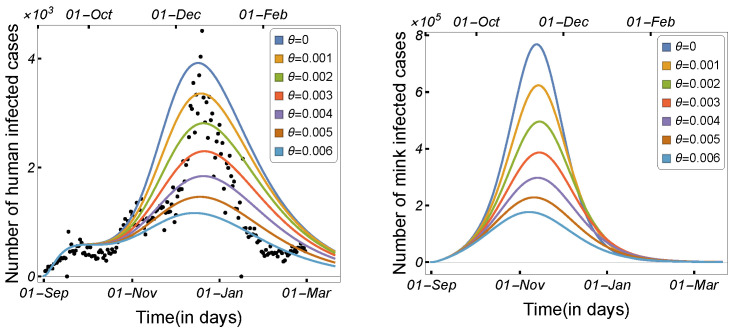
Model (2) solutions (human-infected cases) with respect to the mink vaccination rate (θ): (**left panel**) $I_{u_{1}}$, and (**right panel**) $I_{m_{1}}$, using the parameter values given in Table 2.

The number of daily human and mink infections is shown in Figure 20 in relation to the mink vaccination rate. By increasing the value of θ, the number of COVID-19 cases in humans was significantly reduced, suggesting that the mink vaccine may be beneficial in controlling the epidemic in minks, thereby reducing the number of infected human cases. These findings indicate that the mink vaccination strategy is efficient in controlling the epidemic, although there was no animal vaccine available at the time the virus was introduced in Denmark and transmitted to minks. As a result, rather than killing minks, the vaccination may be sufficient to mitigate the economic impact on mink farming.

#### 3.2.2. The Impact of Culling Minks on the Spread of COVID-19

**Figure 21:**
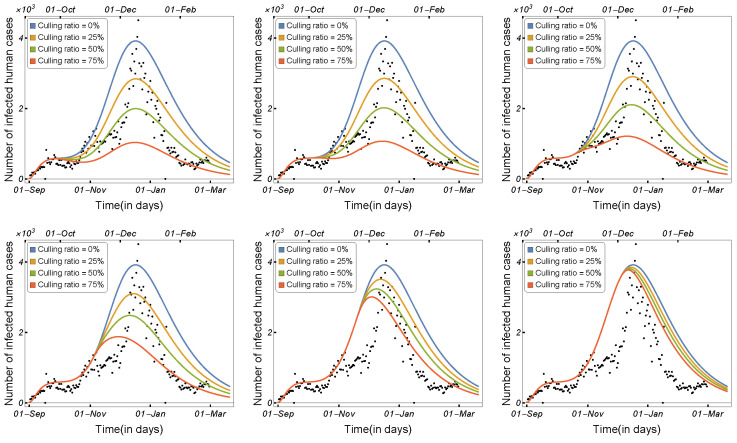
Model (2) solutions (human-infected cases) on the culling dates of 15 September 2020, 1 October 2020, 15 October 2020, 1 November 2020, 15 November 2020, and 1 December 2020, respectively, using the parameters given in Table 2.

**Figure 22:**
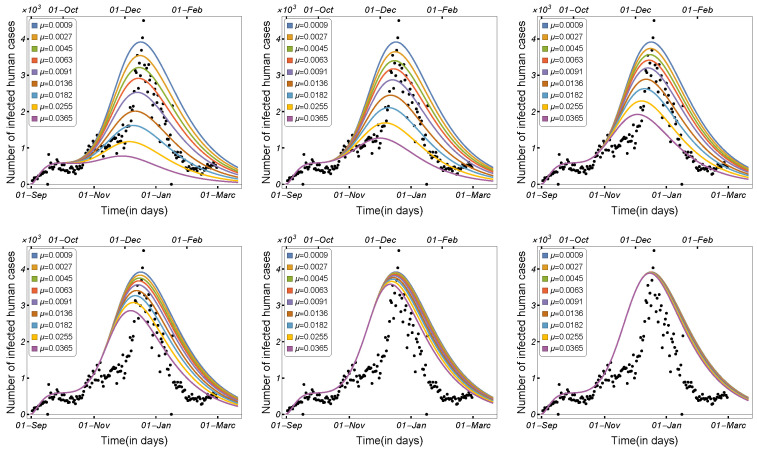
Model (2) solutions (human-infected cases) with respect to the mink death rate on the culling dates of 15 September 2020, 1 October 2020, 15 October 2020, 1 November 2020, 15 November 2020, and 1 December 2020, respectively, using the parameters given in Table 2.

Similar to Figure 18 and Figure 19 in the previous subsection, we applied the single-strain model to investigate the impact of culling minks on COVID-19 virus transmission between humans and minks. Beginning with a culling ratio of zero and continuing until 75% of the population of minks was culled, Figure 21 shows the number of COVID-19 infections in humans before and after mink culling. The effective culling period, according to Figure 21, was between October 2020 and November 2020. These findings, once again, strongly justify Denmark’s plan to euthanize all minks on 4 November 2020. Figure 22 presents the number of COVID-19 infections in humans with respect to the mink death rate. Killing minks early, such as in September and October, was more effective than in November and December. Figure 22 illustrates that the number of COVID-19 infections in humans was reduced after increasing the mink death rate. However, in comparison with Figure 19, achieving these outcomes required much higher mink mortality.

### 3.3. Sensitivity Analysis

**Figure 23:**
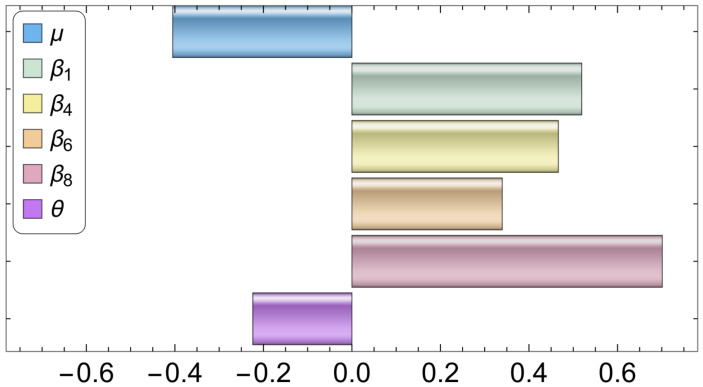
PRCC values describing the relationship between the single-strain model output $I_{u_{1}}+I_{d_{1}}$, and Model (2) parameters $\mu ,\beta_{1},\beta_{4},\beta_{6},\beta_{8}$, and θ. The rest of the parameters are given in Table 2.

Figure 23 displays the correlation between the daily number of infected humans ($I_{u_{1}}+I_{d_{1}}$) and the corresponding one-strain model (2) parameters μ, $\beta_{1},\beta_{4}$, $\beta_{6}$, $\beta_{8}$, and θ that may be subject to intervention measures in COVID-19 control. It is easy to observe that $I_{u_{1}}+I_{d_{1}}$ has a significant positive correlation with $\beta_{1}$, $\beta_{4}$, $\beta_{6}$, and $\beta_{8}$, indicating that an increase in these parameters will increase $I_{u_{1}}+I_{d_{1}}$. Since μ and θ have negative PRCC values, an increase in these parameters will result in a reduction in $I_{u_{1}}+I_{d_{1}}$. According to the results, the human-to-human and mink-to-human transmission rates are the most important factors in SARS-CoV-2 transmission. While mink-to-human transmission ($\beta_{8}$) is relatively less common than human-to-human transmission, it can still contribute to the overall spread of the virus, particularly in settings where minks are raised in close proximity to humans. Additionally, although the rate of mink-to-mink transmission has a lesser effect on the number of infected cases, it has been demonstrated to be a significant factor in SARS-CoV-2 case transmission. Based on these and the results shown in the previous subsections, our findings suggest that both killing minks and establishing a vaccination plan can considerably reduce the number of infected cases.

**Figure 24:**
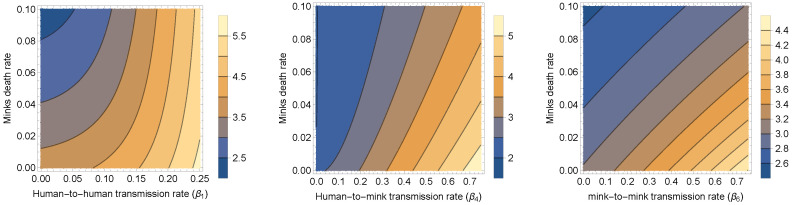
Contour plots of the basic reproduction number $R_{0}$ calculated using the two-strain model (1) as a function of the mink death rate (μ) and the (**left panel**) human-to-human transmission rate ($\beta_{1}$), (**middle panel**) human-to-mink transmission rate ($\beta_{4}$), and (**right panel**) mink-to-mink transmission rate ($\beta_{6}$), using the parameters given in Table 2.

From our compartmental two-strain model, we deduced a formula for the reproduction number. Formula (A3) provides us with the basic reproduction number of Model (1) by substituting the parameter values into it. Using the parameters provided in Table 2 and the formulas in (A1) and (A2), we calculated the values of $R_{1}$ and $R_{2}$ as 1.58432 and 0.565111, respectively. Therefore, the value of the basic reproduction number $R_{0}$ of Model (1) was calculated to be 1.58432. To assess how the basic reproduction number depends on the parameters that can be subject to various intervention measures to control the spread of the SARS-CoV-2 virus, the contour plot of the basic reproduction number is shown as a function of the mink death rate (μ) and human-to-human transmission rate ($\beta_{1}$), human-to-mink transmission rate ($\beta_{4}$), and mink-to-mink transmission rate ($\beta_{6}$) in Figure 24, respectively. The figures clearly indicate that reducing these transmission rates, or at least some of them, has a substantial impact on decreasing the basic reproduction number. Killing minks reduces the transmission rates and hence the number of infected humans. These results support the culling plan launched in Denmark during the large outbreak of the COVID-19 virus in minks.

However, by utilizing the formula in (A4) and the parameter values specified in Table 2, we calculated the basic reproduction number $R_{0}^{V}$ to be 1.71852 based on the model described in Equation (2).

To summarize our findings, we compiled the results obtained in the previous sections in Table 3. These results were obtained by utilizing both the two-strain mathematical model (1) and the single-strain mathematical model (2). Table 3 provides a comprehensive comparison of various factors, including the impact of the transmission rates and the incubation periods, the potential spread of the mutated virus in human or mink populations, the effect of varying mink culling, the influence of mink vaccination, and the values of the basic reproduction number derived using both models applied to the COVID-19 data from Denmark.

## 4. Discussion and Conclusions

COVID-19 is mostly transmitted from person to person, although it has also been known to be transmitted from humans to minks. Mink-to-human transmission, on the other hand, has been documented in the cases of farmed mink in Europe and the United States. In this work, we developed two compartmental models to investigate SARS-CoV-2 virus transmission between humans and minks in Denmark, taking into consideration human-to-human, human-to-mink, mink-to-human, and mink-to-mink transmission of SARS-CoV-2. In the presented new models, we split the human population into two categories based on their level of contact with minks. In the mink population, new SARS-CoV-2 virus strains have been discovered. These variants have been observed to be able to be transmitted back to humans through close contact with infected minks. Therefore, we established a novel two-strain compartmental model, taking into account the possibility of the virus mutation in minks and the spread of the newly mutated virus among humans.

To the best of our knowledge, the models presented in this work are the first compartmental models for SARS-CoV-2 transmission that, in addition to the original virus transmission, take into account the mink mutant strain transmission in both human and mink populations. Our results indicate that if the disease contact rates between humans and minks are high and the disease incubation time is short, this will significantly increase the number of infected minks and, as a result, the overall number of infected humans. We also investigated the possibility that the mutated virus in minks may be transmitted to humans. Moreover, the findings indicate that the mutant strain can invade the original strain under two scenarios: either when the transmission rate is high and the infection period is long, or when the transmission rate and the virus mutation rate are both high. However, whereas mutations in minks increase transmission rates in minks, this does not always translate to increased transmission rates from minks to humans or from humans to humans. Two simulation scenarios are presented to investigate the impact of mink culling on SARS-CoV-2 transmission in mink and humans. The findings support Denmark’s decision to cull approximately 17 million minks on 4 November 2020.

To demonstrate the impact of mink vaccination on SARS-CoV-2 disease transmission between minks and humans, we developed a novel compartmental single-strain model with a mink-vaccinated class. In the absence of an animal vaccine, the findings suggest that the mink vaccination strategy would be effective in suppressing the pandemic, i.e., in decreasing the number of infections in humans. As a consequence, mink vaccination may be another solution instead of killing minks. A sensitivity analysis was carried out to compare the effects of the single-strain model parameters on the number of human-infected cases. We found that the transmission rates from human-to-human and mink-to-human are the most important factors regarding disease transmission. Also, both killing minks and establishing a vaccination plan can considerably reduce the number of infected cases.

Obviously, there are limitations to our models. Concerns that the SARS-CoV-2 mutation might create a risk to human health led to the shutdown of approximately 1500 mink farms in Denmark. Since there are no precise data, or at least no data from all countries that experienced a COVID-19 pandemic in mink farms, on the number of infected minks or mink farms infected with SARS-CoV-2, there are also no precise data on the number of humans who have been affected as a result of the COVID-19 mink mutation. We selected Denmark as an example and utilized our models to study SARS-CoV-2 transmission between minks and humans; however, we found some data in the literature, such as data on the number of mink farm workers or caregivers and the total mink population in Denmark. Unfortunately, there were insufficient data in the literature on SARS-CoV-2 infections in Denmark’s mink farms; thus, we used our models to study SARS-CoV-2 infection in the country’s mink farms with the total mink population, instead of focusing on a specific farm. Due to a lack of data and sources, we found the mink population to be complicated, and it was hard to estimate the appropriate values for different parameters based on the existing literature. Furthermore, we estimated a large number of parameters, which naturally adds complexity because different parameter values may produce equivalent results. Even in cases where multiple parameter sets offered equally good fits, there was a very small difference that enabled us to identify the most optimal one among those that were closely comparable. We conducted extensive research in the literature to acquire the majority of the parameters, and when specific values were unavailable, we determined realistic ranges for these parameters. These ranges formed the foundation for fitting the daily number of COVID-19-infected cases among humans with the most appropriate values. As a result, while there may exist different feasible values within the ranges that yield similar fits, the variations between them should not be significantly distant from one another. Therefore, we determined the best-fit parameter values for COVID-19-infected humans from Denmark, and the best-fit solution is considered as the baseline.

Another limitation of our model was that we were unsure which mink culling strategy was used in Denmark, so we applied mink culling to the whole mink population at the same time, either by using mink culling ratios or increasing the mink death rate. However, Denmark decided to kill all minks by 4 November 2020, and the implementation took some time, which is consistent with our findings. Due to the economic impact on the mink industry, culling minks may not be the best solution. We searched for other possible control measures, such as decreasing mink-to-human transmission by using a single strain model that included a vaccination compartment. Given the lack of an animal vaccine and the uncertainty as to when vaccination should be administered in order to maximize its chances of success, we decided to vaccinate a fraction of the mink population (this is another limitation of our model).

The overall findings of this study suggest that to control the disease and spread of the COVID-19 mutant strains among human and mink populations, we must minimize the transmission and contact rates between mink farmers and other humans through quarantining such individuals. In addition, culling or vaccination strategies for infected mink farms must be implemented in order to reduce the virus mutation rate in minks.

## Figures and Tables

**Table 1:** Descriptions of variables and model parameters for Model (1) and Model (2). Original strain and second-strain variables are differentiated by the lower indices 1 and 2, respectively.

Humans			
**Humans with Indirect Contact**		**Humans with Direct Contact**	
**Variable**	**Description**	**Variable**	**Description**
$S_{u}\left(t\right)$	Susceptible	$S_{d}\left(t\right)$	Susceptible
$E_{u_{1}}\left(t\right),E_{u_{2}}\left(t\right)$	Exposed	$E_{d_{1}}\left(t\right),E_{d_{2}}\left(t\right)$	Exposed
$I_{u_{1}}\left(t\right),I_{u_{2}}\left(t\right)$	Infected	$I_{d_{1}}\left(t\right),I_{d_{2}}\left(t\right)$	Infected
$R_{u}\left(t\right)$	Recovered	$R_{d}\left(t\right)$	Recovered
$D_{u}\left(t\right)$	Death or Removed	$D_{d}\left(t\right)$	Death or Removed
$N_{u}\left(t\right)$	Total population	$N_{d}\left(t\right)$	Total population
**Minks **			
**Variable**	**Description**	**Variable**	**Description**
$S_{m}\left(t\right)$	Susceptible	$R_{m}\left(t\right)$	Recovered
$E_{m_{1}}\left(t\right),E_{m_{2}}\left(t\right)$	Exposed	$D_{m}\left(t\right)$	Death or Removed
$I_{m_{1}}\left(t\right),I_{m_{2}}\left(t\right)$	Infected	V(t), $N_{m}\left(t\right)$	Vaccinated, Total population
**Parameters **			
**Parameter**	**Description**		
$\beta_{1},\beta_{2},\beta_{3}$	Human-to-human transmission rates		
$\beta_{4},\beta_{5}$	Human-to-mink transmission rates		
$\beta_{6},\beta_{7}$	Mink-to-mink transmission rates		
$\beta_{8},\beta_{9}$	Mink-to-human transmission rates		
$\nu_{1},\nu_{2}$	Human incubation rates		
$\nu_{3},\nu_{4}$	Mink incubation rates		
$\sigma_{1},\sigma_{2}$	Human recovery rates		
$\sigma_{3},\sigma_{4}$	Mink recovery rates		
$\delta_{1},\delta_{2}$	Human disease-induced death rates		
Λ, μ	Mink birth and death rates		
$\delta_{3},\delta_{4}$	Mink disease-induced death rates		
γ	Virus mutation rate in minks		
θ, ε	Mink vaccination rate; Rate of infected vaccinated minks		

**Table 2:** Parameters, values, units, and sources of Model (1) and Model (2).

Parameter	Value Model (1)	Value Model (2)	Units	Source
$\beta_{1}$, $\beta_{2}$, $\beta_{3}$	0.104, 0.17, 0.031	0.061, −, −	Day${}^{-1}$	Estimated
$\beta_{4}$, $\beta_{5}$	0.467, 0.02	0.407, −	Day${}^{-1}$	Estimated
$\beta_{6}$, $\beta_{7}$	0.632, 0.02	0.531, −	Day${}^{-1}$	Estimated
$\beta_{8}$, $\beta_{9}$	0.020, 0.02	0.048, −	Day${}^{-1}$	Estimated
$\nu_{1}$, $\nu_{2}$	0.070, 0.23	0.071, −	Day${}^{-1}$	[52,53]
$\nu_{3}$, $\nu_{4}$	0.207, 0.199	0.183, −	Day${}^{-1}$	Estimated
$\sigma_{1}$, $\sigma_{2}$	0.142, 0.118	0.142, −	Day${}^{-1}$	[53]
$\sigma_{3}$, $\sigma_{4}$	0.149, 0.118	0.149, −	Day${}^{-1}$	Estimated
$\delta_{1}$, $\delta_{2}$	0.266, 0.238	0.262, −	Day${}^{-1}$	Estimated
$\delta_{3}$, $\delta_{4}$	0.156, 0.284	0.225, −	Day${}^{-1}$	[54]
Λ, μ	0, 0.0009	0, 0.0009	Day${}^{-1}$	[48,49,50]
γ	0.102	−	Day${}^{-1}$	Estimated
θ, ε	−, −	0, 0.001	Day${}^{-1}$	Estimated
$N_{d}$	2500	2500	Person	[55]
$N_{u}$	5,831,400	5,831,400	Person	[56]
$N_{m}$	17,000,000	17,000,000	Mink	[57]

**Table 3:** Summary of the results obtained using Model (1) and Model (2).

Results Concerning the Two-Strain Model (1) with Mutation
**Impact of transmission rates and incubation periods**
**Parameter** **Total infected humans** human-to-human transmission rates ($\beta_{1},\;\beta_{2},\;\beta_{3}$)large impacthuman-to-mink transmission rate ($\beta_{4}$)little impactmink-to-mink transmission rates ($\beta_{6},\;\beta_{7}$)large impactmink-to-human transmission rates ($\beta_{8},\;\beta_{9}$)large impact **Parameter** **Total infected humans** **Strain 1** **Strain 2** human incubation rate $\nu_{1}$large impactlarge impactlittle impacthuman incubation rate $\nu_{2}$little impactno impactlarge impactmink incubation rate $\nu_{3}$large impactlarge impactlarge impact
**Potential spread of the mutated virus in human or mink populations**
**Scenario 1** Spread was possible in the human populations if the transmission rate ($\beta_{2}$) was high and the infection period ($1/\sigma_{2}$) was long, or when the transmission rate ($\beta_{2}$) and the virus mutation rate (γ) were high **Scenario 2** Spread was possible in human populations if there was a direct increase in the rates of human-to-human transmission ($\beta_{2}$ or $\beta_{3}$) or an increase in the mink-to-human transmission rate ($\beta_{9}$) **Scenario 3** Spread was possible in the mink populations if there was a direct increase in the human-to-mink transmission rate ($\beta_{5}$), the mink-to-mink transmission rate ($\beta_{7}$), or the mink mutation rate (γ)
**The effect of varying the culling of minks**
**Scenario 1** From a culling ratio of 0% to 75% of the mink population, early culling effectively reduced the number of infected humans. The most effective culling period was observed to be from September to November 2020. **Scenario 2** Increasing mink deaths in September and October 2020 led to a significant decrease in COVID-19 cases in humans. However, increasing mink deaths in November and December 2020 had a smaller impact on the number of cases
**Basic reproduction number**
$R_{1}$ $R_{2}$ $R_{0}$
1.58432 0.565111 1.58432
**Results Concerning the Single-Strain Model (2) with Vaccination in Minks**
**Impact of mink vaccination**
**On total infected humans** Increasing the value of the vaccination rate (θ) from 0 to 0.006 had a large impact on decreasing the number of cases. **On total infected minks** Increasing the value of the vaccination rate (θ) from 0 to 0.006 had a large impact on decreasing the number of cases.
**The effect of varying the culling of minks**
**Scenario 1** Beginning with a culling ratio of 0% and continuing until 75% of the mink populations were culled, the effective culling period occurred between October 2020 and November 2020. **Scenario 2** Killing minks early, such as in September and October 2020, was more effective than in November and December 2020.
**Basic reproduction number**
$R_{0}^{V}$ 1.71852

## Data Availability

Not applicable.

## Appendix A. Basic Reproduction Numbers

### Appendix A.1. Basic Reproduction Number of the Two-Strain Model (1)

To calculate the basic reproduction number $R_{0}$ of (1), we follow the general approach established in [44,45]. Given the infectious states $E_{u_{1}}$, $I_{u_{1}}$, $E_{u_{2}}$, $I_{u_{2}}$, $E_{d_{1}}$, $I_{d_{1}}$, $E_{d_{2}}$, $I_{d_{2}}$, $E_{m_{1}}$, $I_{m_{1}}$, $E_{m_{2}}$, and $I_{m_{2}}$ in (1), we can create the transmission vector F representing the new infections arriving only into the exposed compartments, and transition vector V, which includes the movements from the infectious compartments in (1), which are given by

$$F=\left[\begin{array}{c} \beta_{1}\frac{I_{u_{1}}}{N_{u}}S_{u}+\beta_{1}\frac{I_{d_{1}}}{N_{d}}S_{u}\\ 0\\ \beta_{2}\frac{I_{u_{2}}}{N_{u}}S_{u}+\beta_{3}\frac{I_{d_{2}}}{N_{d}}S_{u}\\ 0\\ \beta_{1}\frac{I_{d_{1}}}{N_{d}}S_{d}+\beta_{1}\frac{I_{u_{1}}}{N_{u}}S_{d}+\beta_{8}\frac{I_{m_{1}}}{N_{m}}S_{d}\\ 0\\ \beta_{2}\frac{I_{d_{2}}}{N_{d}}S_{d}+\beta_{3}\frac{I_{u_{2}}}{N_{u}}S_{d}+\beta_{9}\frac{I_{m_{2}}}{N_{m}}S_{d}\\ 0\\ \beta_{4}\frac{I_{d_{1}}}{N_{d}}S_{m}+\beta_{6}\frac{I_{m_{1}}}{N_{m}}S_{m}\\ 0\\ \beta_{5}\frac{I_{d_{2}}}{N_{d}}S_{m}+\beta_{7}\frac{I_{m_{2}}}{N_{m}}S_{m}\\ 0 \end{array}\right],\;\mathrm{and}\;V=\left[\begin{array}{c} \nu_{1}E_{u_{1}}\\ \sigma_{1}I_{u_{1}}-\nu_{1}E_{u_{1}}\\ \nu_{2}E_{u_{2}}\\ \sigma_{2}I_{u_{2}}-\nu_{2}E_{u_{2}}\\ \nu_{1}E_{d_{1}}\\ \sigma_{1}I_{d_{1}}-\nu_{1}E_{d_{1}}\\ \nu_{2}E_{d_{2}}\\ \sigma_{2}I_{d_{2}}-\nu_{2}E_{d_{2}}\\ \nu_{3}E_{m_{1}}++\mu E_{m_{1}}\\ \sigma_{3}I_{m_{1}}+\mu I_{m_{1}}-\nu_{3}E_{m_{1}}\\ \nu_{4}E_{m_{2}}+\mu E_{m_{2}}\\ \sigma_{4}I_{m_{2}}+\mu I_{m_{2}}-\gamma I_{m_{1}}-\nu_{4}E_{m_{2}} \end{array}\right].$$

In the absence of the disease, System (1) has a unique disease-free equilibrium

$$E_{0}=\left(N_{u}^{*},0,0,0,0,0,N_{d}^{*},0,0,0,0,0,N_{m}^{*},0,0,0,0,0\right),$$

where $N_{m}^{*}=\frac{\Lambda }{\mu }$. By substituting the coordinates of $E_{0}$, the matrices *F* and *V* can be obtained for the terms corresponding to new infections and those corresponding to other transfers. We compute the Jacobian *F* from F as

$$\left[\begin{array}{cccccccccccc} 0 & \beta_{1} & 0 & 0 & 0 & \beta_{1}\frac{N_{u}^{*}}{N_{d}^{*}} & 0 & 0 & 0 & 0 & 0 & 0\\ 0 & 0 & 0 & 0 & 0 & 0 & 0 & 0 & 0 & 0 & 0 & 0\\ 0 & 0 & 0 & \beta_{2} & 0 & 0 & 0 & \beta_{3}\frac{N_{u}^{*}}{N_{d}^{*}} & 0 & 0 & 0 & 0\\ 0 & 0 & 0 & 0 & 0 & 0 & 0 & 0 & 0 & 0 & 0 & 0\\ 0 & \beta_{1}\frac{N_{d}^{*}}{N_{u}^{*}} & 0 & 0 & 0 & \beta_{1} & 0 & 0 & 0 & 0 & \beta_{8}\frac{N_{d}^{*}}{N_{m}^{*}} & 0\\ 0 & 0 & 0 & 0 & 0 & 0 & 0 & 0 & 0 & 0 & 0 & 0\\ 0 & 0 & 0 & \beta_{3}\frac{N_{d}^{*}}{N_{u}^{*}} & 0 & 0 & 0 & \beta_{2} & 0 & 0 & 0 & \beta_{9}\frac{N_{d}^{*}}{N_{m}^{*}}\\ 0 & 0 & 0 & 0 & 0 & 0 & 0 & 0 & 0 & 0 & 0 & 0\\ 0 & 0 & 0 & 0 & 0 & \beta_{4}\frac{N_{m}^{*}}{N_{d}^{*}} & 0 & 0 & 0 & \beta_{6} & 0 & 0\\ 0 & 0 & 0 & 0 & 0 & 0 & 0 & 0 & 0 & 0 & 0 & 0\\ 0 & 0 & 0 & 0 & 0 & 0 & 0 & \beta_{5}\frac{N_{u}^{*}}{N_{d}^{*}} & 0 & 0 & 0 & \beta_{7}\\ 0 & 0 & 0 & 0 & 0 & 0 & 0 & 0 & 0 & 0 & 0 & 0 \end{array}\right]$$

and the Jacobian matrix *V* from V as

$$\left[\begin{array}{cccccccccccc} \nu_{1} & 0 & 0 & 0 & 0 & 0 & 0 & 0 & 0 & 0 & 0 & 0\\ -\nu_{1} & \sigma_{1}+\delta_{1} & 0 & 0 & 0 & 0 & 0 & 0 & 0 & 0 & 0 & 0\\ 0 & 0 & \nu_{2} & 0 & 0 & 0 & 0 & 0 & 0 & 0 & 0 & 0\\ 0 & 0 & -\nu_{2} & \sigma_{2}+\delta_{2} & 0 & 0 & 0 & 0 & 0 & 0 & 0 & 0\\ 0 & 0 & 0 & 0 & \nu_{1} & 0 & 0 & 0 & 0 & 0 & 0 & 0\\ 0 & 0 & 0 & 0 & -\nu_{1} & \sigma_{1}+\delta_{1} & 0 & 0 & 0 & 0 & 0 & 0\\ 0 & 0 & 0 & 0 & 0 & 0 & \nu_{2} & 0 & 0 & 0 & 0 & 0\\ 0 & 0 & 0 & 0 & 0 & 0 & -\nu_{2} & \sigma_{2}+\delta_{2} & 0 & 0 & 0 & 0\\ 0 & 0 & 0 & 0 & 0 & 0 & 0 & 0 & \nu_{3}+\mu & 0 & 0 & 0\\ 0 & 0 & 0 & 0 & 0 & 0 & 0 & 0 & -\nu_{3} & \gamma +\sigma_{3}+\mu +\delta_{3} & 0 & 0\\ 0 & 0 & 0 & 0 & 0 & 0 & 0 & 0 & 0 & 0 & \nu_{4}+\mu & 0\\ 0 & 0 & 0 & 0 & 0 & 0 & 0 & 0 & 0 & -\gamma & -\nu_{4} & \sigma_{4}+\mu +\delta_{4} \end{array}\right],$$

hence, we can obtain the characteristic polynomial of $FV^{-1}$ as

$$\lambda^{6}\left(-\lambda^{3}+B\lambda^{2}+C\lambda -D\right)\left(-\lambda^{3}+E\lambda^{2}+F\lambda +G\right)=0,$$

where

$$\begin{array}{cc} B= & \frac{2\beta_{1}}{\left(\sigma_{1}+\delta_{1}\right)}+\frac{\nu_{3}\beta_{6}}{\left(\nu_{3}+\mu \right)\left(\gamma +\sigma_{3}+\delta_{3}+\mu \right)},\\ C= & -\frac{2\nu_{3}\beta_{1}\beta_{6}}{\left(\sigma_{1}+\delta_{1}\right)\left(\nu_{3}+\mu \right)\left(\gamma +\sigma_{3}+\delta_{3}+\mu \right)}+\frac{\nu_{3}\beta_{4}\beta_{8}}{\left(\sigma_{1}+\delta_{1}\right)\left(\nu_{3}+\mu \right)\left(\gamma +\sigma_{3}+\delta_{3}+\mu \right)},\\ D= & \frac{\nu_{3}\beta_{1}\beta_{4}\beta_{8}}{{\left(\sigma_{1}+\delta_{1}\right)}^{2}\left(\nu_{3}+\mu \right)\left(\gamma +\sigma_{3}+\delta_{3}+\mu \right)},\\ E= & \frac{\beta_{2}}{\left(\sigma_{2}+\delta_{2}\right)}+\frac{\nu_{4}\beta_{7}}{\left(\nu_{4}+\mu \right)\left(\sigma_{4}+\delta_{4}+\mu \right)},\\ F= & \frac{\nu_{2}\left(\beta_{3}^{2}-\beta_{2}^{2}\right)}{{\left(\sigma_{2}+\delta_{2}\right)}^{2}}-\frac{2\nu_{4}\beta_{2}\beta_{7}}{\left(\sigma_{2}+\delta_{2}\right)\left(\nu_{4}+\mu \right)\left(\sigma_{4}+\delta_{4}+\mu \right)}+\frac{\nu_{4}\beta_{9}\beta_{5}}{\left(\sigma_{2}+\delta_{2}\right)\left(\nu_{4}+\mu \right)\left(\sigma_{4}+\delta_{4}+\mu \right)}\frac{N_{u}^{*}}{N_{m}^{*}},\\ G= & \frac{\nu_{4}\beta_{7}\left(\beta_{2}^{2}-\beta_{3}^{2}\right)}{{\left(\sigma_{2}+\delta_{2}\right)}^{2}\left(\nu_{4}+\mu \right)\left(\sigma_{4}+\delta_{4}+\mu \right)}-\frac{\nu_{4}\beta_{2}\beta_{5}\beta_{9}}{{\left(\sigma_{2}+\delta_{2}\right)}^{2}\left(\nu_{4}+\mu \right)\left(\sigma_{4}+\delta_{4}+\mu \right)}\frac{N_{u}^{*}}{N_{m}^{*}}, \end{array}$$

First, we deduce the basic reproduction number belonging to the original strain. The characteristic polynomial is given by

$$-\lambda^{3}+B\lambda^{2}+C\lambda -D=0.$$

Thus, the basic reproduction number $R_{1}$ is obtained as the root of the cubic equation

(A1) $$\begin{array}{cc} R_{1}= & \frac{B}{3}+\frac{\sqrt[{3}]{2}\left(B^{2}+3C\right)}{3\sqrt[{3}]{2B^{3}+9BC+27D+3\sqrt{3}\sqrt{4B^{3}D-B^{2}C^{2}+18BCD-4C^{3}+27D^{2}}}}\\ & +\frac{\sqrt[{3}]{2B^{3}+9BC+27D+3\sqrt{3}\sqrt{4B^{3}D-B^{2}C^{2}+18BCD-4C^{3}+27D^{2}}}}{3\sqrt[{3}]{2}}. \end{array}$$

The basic reproduction number of the mutant strain after the virus mutates in the animals can be calculated using the following characteristic equation

$$-\lambda^{3}+E\lambda^{2}+F\lambda +G=0$$

therefore, the basic reproduction number $R_{2}$ of the mutant strain is the root of the cubic equation given by

(A2) $$\begin{array}{cc} R_{2}= & \frac{E}{3}+\frac{\sqrt[{3}]{2}\left(E^{2}+3F\right)}{3\sqrt[{3}]{2E^{3}+9EF+27G+3\sqrt{3}\sqrt{4E^{3}G-E^{2}F^{2}+18EFG-4F^{3}+27G^{2}}}}\\ & +\frac{\sqrt[{3}]{2E^{3}+9EF+27G+3\sqrt{3}\sqrt{4E^{3}G-E^{2}F^{2}+18EFG-4F^{3}+27G^{2}}}}{3\sqrt[{3}]{2}}. \end{array}$$

The dominant eigenvalue of $FV^{-1}$, which represents the basic reproductive number, is given by:

(A3) $$R_{0}=\max\left\{R_{1},R_{2}\right\}.$$

### Appendix A.2. Basic Reproduction Number of the Single-Strain Model (2)

In the absence of the disease, System (1) has a unique disease-free equilibrium given by

$$E_{0}^{v}=\left(N_{u}^{*},0,0,0,,N_{d}^{*},0,0,0,N_{m}^{*},V^{*},0,0,0\right),$$

where $N_{m}^{*}=\frac{\Lambda }{\mu +\theta }$ and $V^{*}=\frac{\theta \Lambda }{\mu (\mu +\theta )}$.

Again, given the infectious states $E_{u_{1}}$, $I_{u_{1}}$, $E_{d_{1}}$, $I_{d_{1}}$, $E_{m_{1}}$, and $I_{m_{1}}$ in (2), we obtain

$$F=\left[\begin{array}{ccccccc} 0 & \beta_{1} & 0 & \beta_{1}\frac{N_{u}^{*}}{N_{d}^{*}} & 0 & 0\\ 0 & 0 & 0 & 0 & 0 & 0\\ 0 & \beta_{1}\frac{N_{d}^{*}}{N_{u}^{*}} & 0 & \beta_{1} & 0 & \beta_{8}\frac{N_{d}^{*}}{N_{m}^{*}}\\ 0 & 0 & 0 & 0 & 0 & 0\\ 0 & 0 & 0 & \beta_{4}\frac{N_{m}^{*}}{N_{d}^{*}} & 0 & \beta_{6}\frac{N_{m}^{*}+\varepsilon V^{*}}{N_{m}^{*}} & \\ 0 & 0 & 0 & 0 & 0 & 0 \end{array}\right]$$

and

$$V=\left[\begin{array}{cccccc} \nu_{1} & 0 & 0 & 0 & 0 & 0\\ -\nu_{1} & \sigma_{1}+\delta_{1} & 0 & 0 & 0 & 0\\ 0 & 0 & \nu_{1} & 0 & 0 & 0\\ 0 & 0 & -\nu_{1} & \sigma_{1}+\delta_{1} & 0 & 0\\ 0 & 0 & 0 & 0 & \nu_{3}+\mu & 0\\ 0 & 0 & 0 & 0 & -\nu_{3} & \delta_{3}+\sigma_{3}+\mu \end{array}\right],$$

therefore, we can calculate the characteristic polynomial of $FV^{-1}$ as

$$\lambda^{3}\left(-\lambda^{3}+B_{1}\lambda^{2}+C_{1}\lambda -D_{1}\right)=0,$$

where

$$\begin{array}{cc} B_{1}= & \frac{2\beta_{1}}{\sigma_{1}+\delta_{1}}+\frac{\nu_{3}\beta_{6}}{\left(\nu_{3}+\mu \right)\left(\delta_{3}+\sigma_{3}+\mu \right)}\frac{N_{m}^{*}+\varepsilon V^{*}}{N_{m}^{*}},\\ C_{1}= & \frac{2\nu_{3}\beta_{1}\beta_{6}}{\left(\sigma_{1}+\delta_{1}\right)\left(\nu_{3}+\mu \right)\left(\delta_{3}+\sigma_{3}+\mu \right)}\frac{N_{m}^{*}+\varepsilon V^{*}}{N_{m}^{*}}-\frac{\nu_{3}\beta_{4}\beta_{8}}{\left(\sigma_{1}+\delta_{1}\right)\left(\nu_{3}+\mu \right)\left(\delta_{3}+\sigma_{3}+\mu \right)},\\ D_{1}= & \frac{\nu_{3}\beta_{1}\beta_{4}\beta_{8}}{{\left(\sigma_{1}+\delta_{1}\right)}^{2}\left(\nu_{3}+\mu \right)\left(\gamma +\sigma_{3}+\mu \right)}. \end{array}$$

Thus, $R_{0}^{V}$ is calculated as the root of the cubic equation $-\lambda^{3}+B_{1}\lambda^{2}+C_{1}\lambda -D_{1}=0$, and is given by

(A4) $$\begin{array}{cc} R_{0}^{V}= & \frac{B_{1}}{3}+\frac{\sqrt[{3}]{2}\left(B_{1}^{2}+3C_{1}\right)}{3\sqrt[{3}]{2B_{1}^{3}+9B_{1}C_{1}+27D_{1}+3\sqrt{3}\sqrt{4B_{1}^{3}D_{1}-B_{1}^{2}C_{1}^{2}+18B_{1}C_{1}D_{1}-4C_{1}^{3}+27D_{1}^{2}}}}\\ & +\frac{\sqrt[{3}]{2B_{1}^{3}+9B_{1}C_{1}+27D_{1}+3\sqrt{3}\sqrt{4B_{1}^{3}D_{1}-B_{1}^{2}C_{1}^{2}+18B_{1}C_{1}D_{1}-4C_{1}^{3}+27D_{1}^{2}}}}{3\sqrt[{3}]{2}}. \end{array}$$
